# Effect of physical activity on patients of NSCLC

**DOI:** 10.1007/s12672-024-01170-2

**Published:** 2024-08-02

**Authors:** Qi Min, Shao Xianru, Sun Gengyun

**Affiliations:** Department of Respiratory and Critical Care Medicine, The First Affiliated Hospital of Anhui Medical University, Hefei, China

**Keywords:** Non-small-cell lung cancer, Immune-related adverse events, Physical activity, Immunotherapy

## Abstract

**Objective:**

The purpose of this study is to assess the impact of physical activity on both therapeutic efficacy and immune-related adverse events (irAEs) during immunotherapy for non-small cell lung cancer (NSCLC).

**Methods:**

Physical activity was divided into three groups: light physical activity (LPA), moderate physical activity (MPA), and vigorous physical activity (VPA) for laboratory indexes, efficacy, and irAEs. A multivariate logistic regression was employed to analyze the relationship between sedentary behavior with efficacy and irAEs.

**Results:**

The study included 121 patients. The three levels of physical activity were not significantly associated with efficacy or irAEs. However, noteworthy disparities were observed in base-hemoglobin levels (F = 3.4, P = 0.037) and base-lymphocyte levels (χ^2^ = 6.13, P = 0.047) among the three groups. After treatment, we identified statistically significant variations in albumin levels (P = 0.012) and lymphocyte counts (P = 0.035). Furthermore, a negative correlation emerged between pre-treatment sedentary behavior duration and immune-efficacy (β: −0.005, P = 0.027).

**Conclusions:**

In summary, within the cohort of NSCLC patients undergoing single immunotherapy or a combination of immunotherapy and chemotherapy, physical activity is closely related to immune and inflammatory indicators in patients, and prolonged sitting will reduce the therapeutic effect.

**Supplementary Information:**

The online version contains supplementary material available at 10.1007/s12672-024-01170-2.

## Introduction

Immune checkpoint inhibitors (ICIs) are a standard of care for non-small cell lung cancer (NSCLC); however, only around 20% of individuals afflicted with lung cancer experience a meaningful response. Regrettably, some lung cancer patients grapple with hyperprogression post-immunotherapy, often accompanied by profound toxicities.

Epidemiological investigations have consistently indicated that physical activity (PA) could reduce the risk of various cancer types [1], curb cancer recurrence, and diminish mortality rates [2]. An accumulation of research underscores the immunomodulatory potential of exercise, particularly to enhance the effectiveness of immunotherapy [3, 4]. HI AIM is the first project to combine supervised and monitored exercise in patients with lung cancer, accompanied by rigorous analyses of immune and cancer cell markers throughout the duration of the trial [5]. The gathered data holds promising implications for endorsing exercise as a strategic modality to mobilize immune system cells, thereby enhancing the efficacy of immunotherapy. Additionally, multiple studies in rodent models have consistently affirmed the synergistic relationship between exercise and cancer immunotherapy [6, 7].

Yo Kawaguchi introduced the concept of the "lung cancer-skeletal muscle cycle" as a negative prognostic loop [8]. He emphasized the potential of PA to ameliorate skeletal muscle loss, consequently elevating myokines levels and impeding tumor growth. In patients surgically treated for NSCLC, postoperative PA interventions exhibit the capability to enhance exercise capacity and respiratory muscle strength following lung resection [9]. For individuals with advanced lung cancer, Temel et al. explored the viability and impacts of physical exercise [10]. Among the 25 patients who completed the structured training regimen, a notable reduction in lung cancer symptoms was observed, with no accompanying decline in muscle strength. Furthermore, engaging in physical activity prior to the initiation of lung adenocarcinoma treatment demonstrates a capacity to prolong the time until the deterioration of Health-related quality of life [11]. In addition, numerous studies have posited a negative correlation between sarcopenia and the effectiveness of immunotherapy [12].

Nonetheless, the influence of physical activity on the immunotherapy effect of NSCLC remains enigmatic. Therefore, our study delves into the connections between physical activity and immunotherapy efficacy, irAEs, as well as biochemical parameters and blood routine in NSCLC.

## Participants and methods

### Study population

We enrolled individuals diagnosed with non-small cell lung cancer, who sought care at the First Affiliated Hospital of Anhui Medical University between August 2022 and February 2023. Inclusion criteria encompassed: (1) The diagnosis was clear and patient received treatment for the first time in our hospital; (2) Patient was receiving either single immunotherapy or a combination of immunotherapy and chemotherapy, and had completed a minimum of four treatment cycles; (3) Patients demonstrating adequate organ function with an Eastern Cooperative Oncology Group Performance Status (ECOG PS) of 0 or 1; and (4) Absence of any contraindications to physical activity and cognitive impairment, thereby allowing for the successful completion of the International Physical Activity Questionnaire (IPAQ).

The ethics committee of the First Affiliated Hospital of Anhui Medical University approved the study (Quick-PJ2024-01-42).All methods adhered to the pertinent guidelines and regulations. All data was obtained with the informed consent of the patient. Due to the observational nature of this study, the ethics committee granted an exemption for printed informed consent.Physical activity assessment.

We employed the abbreviated version of the IPAQ to assess physical activity [13, 14]. This instrument assigned metabolic equivalent of task (MET) ratings of 3.3 for walking, 4.0 for moderate-intensity activity, and 8.0 for high-intensity activity. Data integrity was ensured through rigorous data cleaning and outlier exclusion protocols.

We converted daily activity time into minutes for each activity. To account for a standard eight hours of sleep daily for each participant, individuals were disqualified from the analysis if their cumulative daily activity time across the three categories exceeded 960 min (equivalent to 16 h).

Recognizing that a minimum of ten minutes of continuous physical activity per session is required for health benefits, any reported daily cumulative duration of intensity below this threshold was recorded as "0," along with its corresponding weekly frequency. Moreover, in instances where the daily duration of a specific intensity of physical activity surpassed 3 h, it was capped at 180 min. This protocol allowed for a maximum reporting of 21 h (1260 min) of physical activity per week, preventing erroneous classification of individuals into the "high" activity group. Infrequent, isolated bursts of activity do not yield the same health benefits as consistent, sustained high levels of physical activity.

The weekly quantification of an individual's engagement in a specific intensity of physical activity was determined by the product of the MET value assigned to the activity, the weekly frequency of engagement, and the daily duration of the activity. Following the guidelines set forth by the IPAQ working group (refer to the attachment), individual physical activity levels were categorized into three groups: VPA, MPA, and LPA.

### Blood samples and efficacy evaluation

We gathered questionnaire responses, along with blood routine and biochemical indicators, from patients at both their initial and fifth admissions. Subsequently, we conducted a comprehensive four-month follow-up to assess the occurrence of irAEs.

We computed the neutrophil-to-lymphocyte ratio (NLR) and the platelet-to-lymphocyte ratio (PLR) using the following hematological parameters: absolute neutrophil count, absolute lymphocyte count, and platelet count. Patient outcomes were evaluated after the completion of four treatment cycles, with " complete response (CR)," " partial remission (PR)," and " stable disease (SD)" defined as indicative of treatment effectiveness, while " progressive disease (PD)" signified treatment ineffectiveness.

### Statistics analysis

We employed SPSS 25.0 software for statistical analysis in this study. The baseline characteristics, hematological markers, and irAEs of patients with varying levels of physical activity were scrutinized using T-tests and χ^2^ tests. Logistic regression was used to analyze the relationship between clinical, hematological markers, PA, curative effect, and irAEs. If the P-value on both sides was below 0.05, the difference was considered statistically significant.

Result.

### Patients and basic information

A total of 121 patients were enrolled in the study, 89% of whom were male. Most of the patients were treated with anti-programmed cell death protein-1 (PD-1) antibody (42 patients with tislelizumab, 38 patients with pembrolizumab, 20 patients with camrelizumab, and 9 patients with sintilimab) combined with platinum and paclitaxel or pemetrexed chemotherapy (90%), a few patients were treated with immunotherapy alone or PD-L1 therapy. Patients were assigned into one of the three pre-treatment activity groups: light (n = 17), moderate (n = 27), and vigorous (n = 77) physical activity. Table 1 shows that the mean age of the three groups was 66 years. No statistical difference was found in pathological type, treatment, or tumor stage among the three groups.

**Table 1:** Comparison of different levels of physical activity before treatment

	LPA (N = 1714.1%)	MPA (N = 2722.3%)	VPA (N = 7763.6%)	F/χ^2^	P
Sex: Male	14 (13%)	25 (23.1%)	69 (63.9%)	1.17	0.558
Female	3 (23.1%)	2 (15.4%)	8 (61.5%)		
Age	66 (62.70)	66 (63, 70)	66 (64, 68)	0.01	0.993
Pathogenic type: squama cancer	7 (10.1%)	13 (18.8%)	49 (71.0%)	3.98	0.136
adenocarcinoma	10 (19.2%)	14 (26.9%)	28 (53.8%)		
Grades: stage of III	6 (14.3%)	8 (19.0%)	28 (66.7%)	0.40	0.818
stage of IV	11 (13.9%)	19 (24.1%)	49 (62%)		
therapeutics: immunotherapy	0 (0.0%)	0 (0.0%)	7 (100%)	4.25	0.12
Chemicotherapy + immunotherapy	17 (14.9%)	27 (23.7%)	70 (61.4%)		
irAEs: No	14 (13.2%)	25 (23.6%)	67 (63.2%)	1.08	0.584
Yes	3 (20.0%)	2 (13.3%)	10 (66.7%)		
therapeutic efficacy: SD + PR + CR	12 (12.9%)	19 (20.4%)	62 (66.7%)	1.60	0.45
PD	5 (17.9%)	8 (28.6%)	15 (53.6%)		

### The difference in laboratory indexes in different physical activity groups

**Fig. 1:**
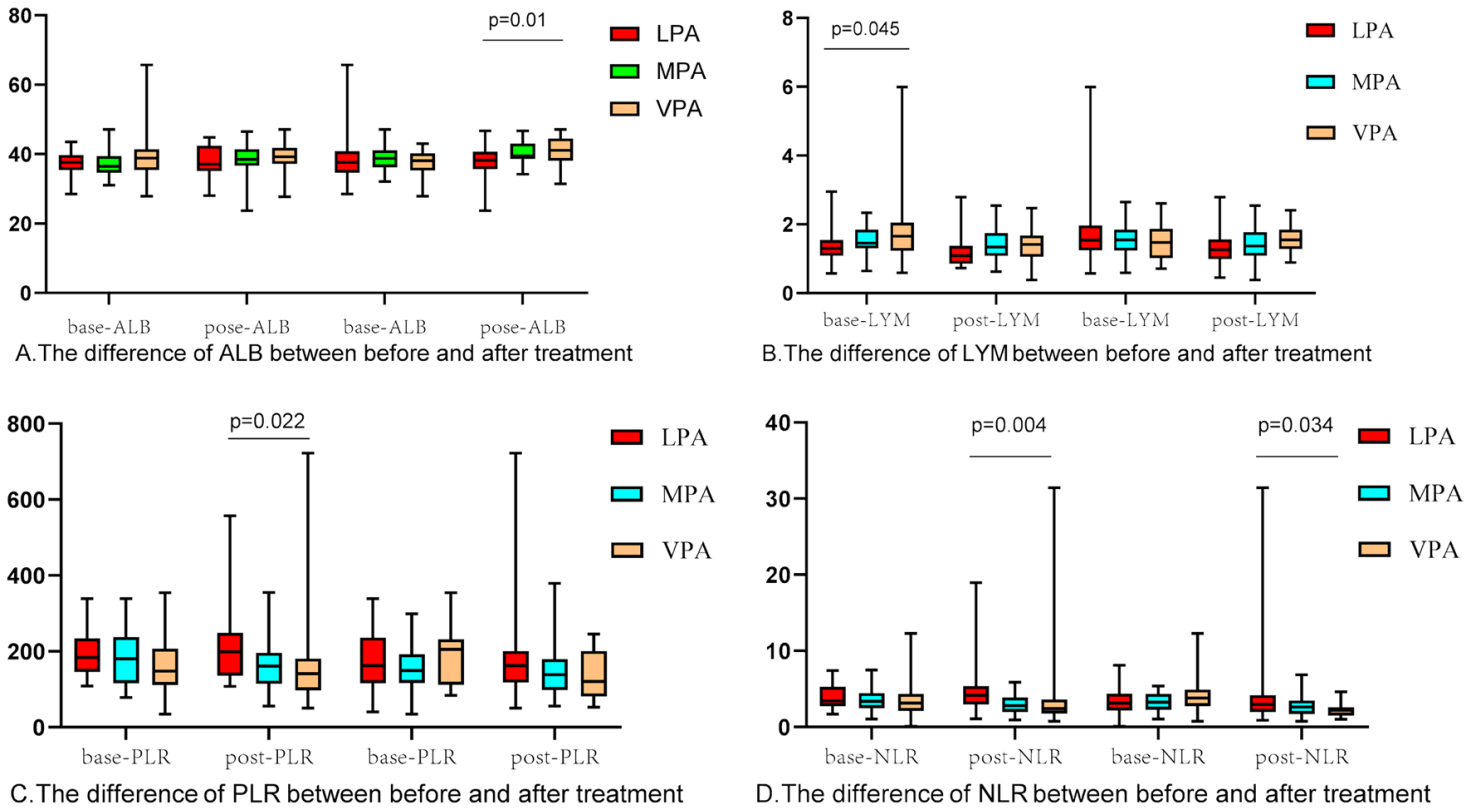
The differences of albumin, lymphocytes, PLR and NLR between different exercise groups before treatment and the differences of albumin, lymphocytes, PLR and NLR between different exercise groups after treatment were analyzed. The differences of the baseline values and the values after treatment in each group were analyzed

**Fig. 2:**
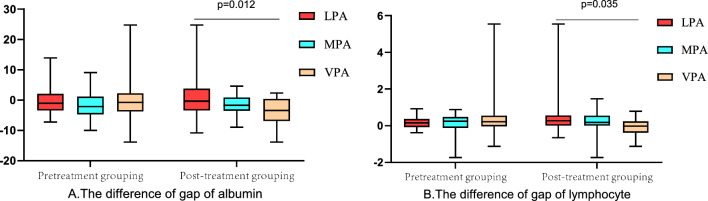
Analysis of gap of albumin and lymphocytes between different exercise groups before treatment and between different exercise groups after treatment

In the baseline physical activity group, we observed a statistical difference in lymphocyte count (P = 0.047) among the three levels of physical activity (Fig. 1). The higher physical activity group showed higher lymphocyte count and hemoglobin levels. In the physical activity group after treatment, there were significant differences in albumin concentration among the three physical activity groups (P = 0.01) (Fig. 1), and there were also statistically significant changes in albumin and lymphocytes after treatment (Fig. 2).

### Evaluation of efficacy and immune adverse reactions in different physical activity groups

In the efficacy evaluation, 28 patients had disease progression, 58 patients had stable disease, 34 patients had partial remission, and 1 case had complete remission. In addition, a total of 15 patients were found to have adverse immune reactions during treatment and follow-up, including 6 cases of immune-related pneumonia, 4 cases of hypothyroidism, 4 cases of rash, and 1 case of immune-related thrombocytopenia.

We did not observe statistically significant differences related to immune efficacy and adverse immune events in either the baseline physical activity group or the post-treatment physical activity group. We performed a binary logistic regression analysis to assess the relationship between pre- and post-treatment physical activity and treatment outcomes, and the gender of the patient, treatment mode, irAEs, laboratory indexes thought to be related to the efficacy were considered as covariables. The results showed that basal sedentary time was negatively correlated with the therapeutic effect (P = 0.027) (Table 2), that is, longer basal sedentary time may be correlated with poorer therapeutic outcomes.

**Table 2:** Multivariate binary Logistic regression analysis of curative effect

	Before treatment			After treatment		
	β	Exp(B)(95%)	P	β	Exp(B)(95%)	P
Sex:Male	−0.609	0.544(0.083,3.579)	0.526	0.097	1.102(0.181,6.704)	0.916
irAEs:No	−1.302	0.272(0.049,1.523)	0.139	−1.364	0.256(0.044,1.473)	0.127
therapeutics: immunotherapy	−1.296	0.274(0.029,2.542)	0.254	−1.356	0.258(0.028,2.347)	0.229
base-Albumin	0.134	1.143(0.98,1.334)	0.088	0.108	1.114(0.958,1.294)	0.16
base-IBIL	0.377	1.458(1.08,1.967)	0.014	0.31	1.364(1.043,1.784)	0.024
base-DBIL	−0.163	0.85(0.582,1.241)	0.4	−0.056	0.945(0.668,1.338)	0.751
base-HB	−0.026	0.974(0.939,1.011)	0.165	−0.032	0.968(0.933,1.005)	0.087
base-PLR	0.004	1.004(0.994,1.014)	0.421	0.006	1.006(0.996,1.015)	0.253
base-NLR	−0.117	0.89(0.631,1.255)	0.505	−0.188	0.829(0.586,1.172)	0.288
Vigorous PA days	0.065	1.068(0.827,1.378)	0.616	0.19	1.209(0.818,1.787)	0.34
Duration of vigorous activity	0.002	1.002(0.997,1.007)	0.455	0.005	1.005(0.991,1.02)	0.486
Moderate PA days	−0.048	0.953(0.765,1.188)	0.668	0.192	1.211(0.851,1.725)	0.288
Duration of moderate activity	0	1(0.995,1.006)	0.877	0.001	1.001(0.989,1.013)	0.872
Walking days	0.351	1.421(0.956,2.111)	0.082	0.004	1.004(0.764,1.318)	0.978
Duration of walking	−0.012	0.989(0.965,1.012)	0.341	0.004	1.004(0.984,1.025)	0.712
Sedentary time	−0.005	0.995(0.991,0.999)	0.027	0.001	1.001(0.998,1.004)	0.509
Intercept	−0.893	0.409	0.777	-0.983	0.374	0.738

When adverse immune events were taken as dependent variables, patients' age, gender, pathological type, treatment mode, basic laboratory indexes, and detailed activities before and after treatment were determined to be covariables. The results showed that basic PLR was negatively correlated with the occurrence of adverse immune events (P = 0.04) (Table 3).

**Table 3:** Multivariate binary Logistic regression analysis of irAEs

	Before treatment			After treatment		
	β	Exp(B)(95%)	P	β	Exp(B)(95%)	P
Sex:Male	−1.762	0.172(0.022,1.345)	0.093	−1.589	0.204(0.029,1.42)	0.108
Age	0.044	1.045(0.955,1.145)	0.338	0.044	1.045(0.971,1.125)	0.238
therapeutics: immunotherapy	1.253	3.5(0.21,58.293)	0.383	0.448	1.566(0.088,27.845)	0.76
Pathogenic type:squama cancer	0.194	1.214(0.317,4.648)	0.777	0.257	1.293(0.326,5.124)	0.715
base-Albumin	−0.027	0.973(0.813,1.166)	0.769	−0.001	0.999(0.855,1.167)	0.994
base-IBIL	−0.345	0.708(0.483,1.039)	0.078	−0.243	0.784(0.562,1.093)	0.151
base-DBIL	0.224	1.251(0.804,1.947)	0.32	0.198	1.219(0.823,1.806)	0.323
base-HB	0.017	1.017(0.97,1.067)	0.48	0.007	1.007(0.963,1.053)	0.764
base-NLR	0.323	1.381(0.881,2.165)	0.159	0.329	1.39(0.898,2.15)	0.139
base-PLR	−0.015	0.985(0.971,0.999)	0.039	−0.013	0.988(0.974,1.001)	0.075
Vigorous PA days	−0.186	0.83(0.582,1.185)	0.306	−0.239	0.788(0.47,1.319)	0.364
Duration of vigorous activity	−0.004	0.996(0.987,1.004)	0.324	−0.005	0.995(0.976,1.015)	0.639
Moderate PA days	0.009	1.009(0.753,1.352)	0.951	0.027	1.028(0.743,1.422)	0.868
Duration of moderate activity	0.002	1.002(0.995,1.01)	0.569	0	1(0.987,1.013)	0.982
Walking days	0.008	1.008(0.66,1.538)	0.972	0.081	1.085(0.693,1.698)	0.723
Duration of walking	−0.008	0.992(0.962,1.023)	0.6	−0.025	0.975(0.921,1.033)	0.391
Sedentary time	0.002	1.002(0.997,1.008)	0.341	−0.003	0.997(0.993,1.001)	0.133
Intercept	−1.428	0.24	0.809	−1.198	0.302	0.808

## Discussion

In our investigation, we conducted a comprehensive assessment of disparities in laboratory indexes, curative effect, and irAEs among patients with varying levels of physical activity both pre- and post-treatment. Patients with higher levels of physical activity before treatment had higher levels of hemoglobin and lymphocytes, and those with higher levels of physical activity after treatment had higher levels of albumin and lower levels of NLR. Despite the discernible variations in immune and nutritional statuses across different physical activity levels, we did not discern any statistically significant associations between physical activity with the curative effect and irAEs. However, our logistic regression analysis unveiled an intriguing finding: A longer duration of sedentary behavior before treatment correlated with a less favorable treatment outcome. This observation suggests a potential link between pre-treatment sedentary lifestyle and diminished treatment efficacy, warranting further investigation.

In our study, the distribution of PA levels among participants was 14.1%, 22.3%, and 63.6%, respectively. This closely with the findings of a study examining the correlation between physical activity and stroke in a cohort of 10,398 middle-aged and older Chinese individuals [15], which reported rates of 15.5%, 26.7%, and 57.8%, respectively. A nationwide survey assessing the prevalence of Physical Inactivity (PI) [16], computed based on the proportion of LPA, in middle-aged and elderly populations revealed that the prevalence rates for men and women were 17.4% and 20.7%, respectively. Additionally, an investigation into physical activity patterns across eastern, central, and western regions of China demonstrated variations in PA levels among diverse ethnic groups and genders [17]. Consequently, the likelihood of selection bias in our study appears to be low, given the observed regional and demographic distinctions in PA. Despite the well-documented decline in physical function among individuals undergoing chemotherapy for lung cancer during the initial stages [18], an array of impediments hinders the engagement of these patients in PA [19]. Within the scope of this investigation, a mere seven individuals received exclusive immunotherapy, and the variance in treatment regimens demonstrated a marginal impact on post-treatment physical activity (The post-treatment PA groups are delineated in Supplemently Table 3.).

The effect of exercise on lymphocytes is complex and is affected by the intensity, duration, and frequency of exercise. We focused on assessing an individual's physical activity, rather than specific planned training, and our results showed that the lymphocytes of the high physical activity group were higher, which was inconsistent with previous studies [20, 21]. Most studies on lymphocytes and exercise have indicated that although vigorous exercise inhibits lymphocyte transformation responses, no changes in lymphocyte count have been observed [22]. However, one study showed that 12 weeks of aerobic exercise increased lymphocyte count, suggesting that exercise leads to an increase in lymphocyte count [23], consistent with our observations. This indicates that exercise has a positive effect on lymphocytes.

It is important to highlight that post-treatment lymphocyte counts have no relationship with physical activity. This observation may be attributed to the fact that a substantial number of patients received concurrent chemotherapy, a treatment known to induce myelosuppression [24]. Furthermore, the malignancy itself may exert inhibitory influences on bone marrow hematopoietic processes, potentially confounding the observed impact of physical activity. Nevertheless, our investigation did reveal a noteworthy finding: The high physical activity group exhibited a lower NLR, consistent with prior research findings [25]. Exercise has been associated with a reduction in neutrophil levels [20], and although statistical significance was not achieved in our study, a discernible trend was evident (P = 0.059). Thus, the decreased neutrophil counts and increased lymphocyte counts reduced the NLR level.

Several studies highlighted the influence of host inflammation levels on the effectiveness of immunotherapy, with some inflammatory markers serving as predictive indicators of immunotherapy response [26]. We observed that physical activity impacted the inflammatory status of lung cancer patients. However, we did not observe a significant relationship between physical activity and therapeutic effects. Despite the well-established associations between inflammation, cancer outcomes, and adverse events, our findings suggest that physical activity may have a limited direct impact. A prospective cohort study reported that physical activity and white blood cell count are independent risk factors for cancer, but did not substantiate inflammation as a mediating factor in the relationship between physical activity and cancer risk [27].

In our logistic regression analysis, an intriguing result emerged: Increased sedentary time was negatively correlated with treatment efficacy, potentially attributed to heightened inflammation levels in sedentary patients. A cross-sectional study identified a positive association between sedentary time and elevated levels of interleukin-6 and C-reactive protein [28]. The sedentary, particularly when it was observed in the pretreatment period might be a surrogate of sarcopenia and weight loss, both features known to be a factor of poor response to ICI [12]. In addition, sedentary patients may have more underlying diseases, and their performance status is poor, leading to poor prognosis. Performance status is also an important factor with poor performance patients having been observed to be affected by a poor patient's outcome when receiving ICI [29]. Furthermore, we found a negative correlation between adverse immune reactions and baseline PLR, consistent with prior research [30], PLR could emerge as an independent predictor of IRAEs. Physical activity was not associated with irAEs, same as efficacy. This may be attributed to the multifaceted nature of risk factors contributing to irAEs [31].

We noted a positive association between physical activity and albumin levels. Albumin, a key biochemical indicator, is susceptible to nutritional status and acute inflammation [32, 33]. The established correlation between physical activity and albumin levels aligns with the fact that exercise stimulates albumin synthesis [34]. Consequently, individuals engaging in vigorous physical activity tend to exhibit higher albumin levels. Moreover, heightened physical activity is linked to reduced chronic inflammation, while individuals with lower physical strength levels often experience elevated inflammatory cytokines, which can trigger a catabolic state, muscle proteolysis, and hinder muscle protein mass recovery [35]. Additionally, malnourished individuals, characterized by depleted protein reserves and diminished muscle mass, tend to exhibit lower physical activity levels. Although albumin level was not linked to treatment efficacy, it is worth noting that malnutrition is a recognized prognostic indicator for patients with NSCLC, a consensus held by both oncologists and nutrition experts [36].

This study offers valuable insights into the interplay between physical activity and immunotherapy of NSCLC. Nevertheless, our study has some limitations that warrant consideration. Firstly, the reliance on self-reported daily activity data obtained through questionnaires represents a crude measurement approach. Secondly, the study's single-center design and relatively small sample size underscore the necessity for a multicenter study to validate these results.

Within the cohort of NSCLC patients undergoing single immunotherapy or a combination of immunotherapy and chemotherapy, physical activity demonstrated a positive impact on alleviating the inflammatory status of patients, while sedentary individuals exhibited notably diminished treatment efficacy. Given its cost-effectiveness, physical activity serves as an auxiliary measure, and we recommend patients engage in moderate exercise and avoid a sedentary lifestyle.

### Supplementary Information

Additional file 1.

Additional file 2.

## Data Availability

The authors will supply the relevant data in response to reasonable requests.

## Abbreviations

irAEs: Immune-related adverse events
NSCLC: Non-small cell lung cancer
LPA: Light physical activity
MPA: Moderate physical activity
VPA: Vigorous physical activity
PA: Physical activity
ICIs: Immune checkpoint inhibitors
IPAQ: International Physical Activity Questionnaire
MET: Metabolic equivalent of task
NLR: Neutrophil-to-lymphocyte ratio
PLR: Platelet-to-lymphocyte ratio
CR: Complete response
PR: Partial remission
SD: Stable disease
PD: Progressive disease
PI: Physical Inactivity

## Declarations

Ethics approval and consent to participate.

The questionnaire and methodology for this study was approved by the Human Research Ethics committee of Anhui Medical University(Quick-PJ2024-01-42).
