# Supplementary material for: Effect of physical activity on patients of NSCLC
Source: Discov Oncol. 2024 Aug 2;15:328. doi: 10.1007/s12672-024-01170-2 (PMC11297224; doi:10.1007/s12672-024-01170-2)
Supplement: Supplementary file 1 — Additional file 1. [file 12672_2024_1170_MOESM1_ESM.docx]

| Supplemently Table 1 Physical activity attributes and MET values in IPAQ | | | |
| --- | --- | --- | --- |
| Type | Item | Strengh of physical activity | MET value |
| Work-related | Walk | Walk | 3.3 |
|  | Moderate-strength | Moderate | 4.0 |
|  | High-strength | High | 8.0 |
| Transportation-related | Walk | Walk | 3.3 |
|  | Ride | Moderate | 6.0 |
| Household-related | Moderate-streng outdoor housework | Moderate | 3.0 |
|  | Moderate-strength indoor housework | Moderate | 4.0 |
|  | High-strength outdoor housework | Moderate | 5.5 |
| Leisure-related | Walk | Walk | 3.3 |
|  | Moderate-strength | Moderate | 4.0 |
|  | High-strength | High | 8.0 |

| Supplemently Table 2 Standardize of physical activity level | |
| --- | --- |
| Physical activity level | Standardization |
| High-level | Meet any of the following 2 criteria: |
|  | 1. All kinds of high-strength physical activity were carried out ≥ 3 days, and the weekly total physical activity level ≥1 500 met-min /w |
|  | 2. The three levels of physical activity totaled ≥ 7 days, and the total weekly physical activity level ≥ 3 000 met-min /w |
| Moderate-level | Meet any of the following 3 criteria: |
|  | 1. All kinds of high-strength physical activity for at least 20 minutes per day≥3 days |
|  | 2. At least 30 minutes of moderate-strength and/or walking activity per day ≥ 5 days |
|  | 3. The three levels of physical activity were carried out ≥ 5 days, and the total weekly physical activity level ≥ 600 met-min /w |
| Low-level | Meet any of the following 2 criteria: |
|  | 1. No physical activity was reported |
|  | 2. Some activities were reported but did not meet the above standardization of moderate or high-level |
