# Supplementary material for: Effect of physical activity on patients of NSCLC
Source: Discov Oncol. 2024 Aug 2;15:328. doi: 10.1007/s12672-024-01170-2 (PMC11297224; doi:10.1007/s12672-024-01170-2)
Supplement: Supplementary file 2 — Additional file 2. [file 12672_2024_1170_MOESM2_ESM.docx]

| Supplemently Table 3. Comparison of different levels of physical activity after treatment | | | | | |
| --- | --- | --- | --- | --- | --- |
|  | LPA | MPA | VPA | F/χ^2^ | P |
| Sex: Male | 68（63.0%） | 25（23.1%） | 15（13.9%） | 1.59 | 0.452 |
| Female | 7（53.8%） | 5（38.5%） | 1（7.7%） |  |  |
| Age | 67（64，69） | 65（62，68） | 67（63，71） | 0.50 | 0.609 |
| Pathogenic type: squama cancer | 46（66.7%） | 11（15.9%） | 12（17.4%） | 7.75 | **0.021** |
| adenocarcinoma | 29（55.8%） | 19（36.5%） | 4（7.7%） |  |  |
| Grades: stage of III | 30（71.4%） | 6（14.3%） | 6（14.3%） | 3.85 | 0.146 |
| stage of IV | 45（57%） | 24（30.4%） | 10（12.7%） |  |  |
| therapeutics：ICI | 5（71.4%） | 2（28.6%） | 0（0.0%） | 1.32 | 0.568 |
| ICI+CT | 70（61.4%） | 28（24.6%） | 16（14.0%） |  |  |
